# Complete chloroplast genome of the medicinal herb *Veronicastrum axillare* (Sieb. et Zucc.) Yamazaki and the phylogenetic relationship analysis within the tribe Veroniceae

**DOI:** 10.1080/23802359.2022.2071651

**Published:** 2022-05-05

**Authors:** Jiawen Luo, Qiuyi Gong, Manjia Zhou, Qiang Liu, Rubin Cheng, Yuqing Ge

**Affiliations:** a The First Affiliated Hospital, Zhejiang Chinese Medical University, Hangzhou, China; b School of Pharmaceutical Sciences, Zhejiang Chinese Medical University, Hangzhou, China; c Academy of Chinese Medical Science, Zhejiang Chinese Medical University

**Keywords:** *Veronicastrum axillare*, tribe Veroniceae, complete chloroplast genome, phylogenetic analysis

## Abstract

*Veronicastrum axillare* (Sieb. et Zucc.) Yamazaki is a traditional medical plant with versatile biological activities. Here, we reported the complete chloroplast genome sequence of *V. axillare* with a total length of 152,691 bp, containing two IR regions of 25,765 bp each, an LSC region of 83,559 bp, and an SSC region of 17,602 bp. The genome encodes 131 genes, including 85 protein-coding genes, 37 tRNAs, eight rRNAs, and one pseudogene (*ycf1*). The overall GC content is 38.3%, with the highest content of 43.31% in IR region. Comparative analysis revealed 4 potential hotspots among *V. axillare* and other Veroniceae plants, providing potential markers for population investigations in the tribe Veroniceae. A total of 56 simple sequence repeats were identified in *V. axillare.* Phylogenetic analysis indicated a sister relationship between *V. axillare* and *V. sibiricum*, suggesting a close genetic relationship between the two *Veronicastrum* species. Our results provide basic genetic resources for investigating the evolutionary status of *V. axillare* within the tribe Veroniceae.

*Veronicastrum axillare* (Sieb. et Zucc.) Yamazaki 1957 is a perennial herb, which belongs to the tribe Veroniceae of the family Plantaginaceae. It has bent stems, hairless paper-like leaves, and axillary inflorescence. Although this species is primarily found in the grasslands of the mountainous regions of southeastern China, it has also spread to Japan (Zheng et al. 2014). *V. axillare* has been used as a folk medicine named ‘Fushuicao’ to treat nephritic edema, cirrhotic ascites, venomous snakebite, and furuncles (Deng et al. 2014). Due to the paraphyletic subgenera and the lack of genome support, the phylogenetic relationships of the tribe Veroniceae were poorly understood (Albach and Meudt 2010). The complete chloroplast (cp) genome sequences of the four Veroniceae species provided valuable data for the elucidation of the classification and taxa delimitation among the Veroniceae plants (Choi et al. 2016; Maurya et al. 2020). The cp genome of *V. axillare* showed the sequenced fifth chloroplast genome in the tribe Veroniceae, resulting in the development of effective molecular markers for the *Veronicastrum* genus and the taxonomic relationships within the tribe Veroniceae.

We collected the *V. axillare* leaf sample from the Botanical Garden of Medicinal Plants of the Zhejiang Chinese Medical University in the Fuyang area of Zhejiang Province, China (30°05′2.4″ N, 119°53′20.4″ E). We submitted the leaf specimen at the Medicinal Herbarium Center of the Zhejiang Chinese Medical University (https://yxy.zcmu.edu.cn; Herbarium Code: MHCZCMU; Collector: Rubin Cheng, biothcheng@hotmail.com) under the voucher number PYH-2019032. We sequenced the extracted total genomic DNA using the Illumina Hiseq Platform according to our previous reports (Dong et al. 2021; He et al. 2021). In order to obtain the chloroplast genome of *V. axillare*, we trimmed the raw reads using Trimmomatic and assembled the resulting cleaned reads using metaSPAdes 3.13.0 with the cp genome of the homologous species of *Veronicastrum sibiricum* (NC_031345) as the reference genome (Choi et al. 2016). We used GeSeq to annotate the chloroplast genome and further confirmed it using BLAST (Tillich et al. 2017). We submitted the complete cp genome of *V. axillare* to GenBank (Accession Number: MW244757).

The length of the complete chloroplast (cp) genome of *V. axillare* was 152,691 bp, with a conserved quadripartite structure containing an LSC region (83,559 bp), an SSC region (17,602 bp), and two IR regions of 25,765 bp each. We identified 131 genes in *V. axillare*, including 85 protein-coding genes, 37 tRNAs, eight rRNAs, and one pseudogene (*ycf1*). The estimated overall base composition of the *V. axillare* cp genome was A (30.46%), C (19.44%), G (18.86%), and T (31.23%), thus exhibiting an obvious AT-richness (61.69%). The cp genome of *V. axillare* contained 16 duplicated genes in the IR region, including seven tRNAs, four rRNAs, and five protein-coding genes. The most frequently used amino acids were Leu (10.65%), followed by Ile (8.42%), Ser (7.93%), Gly (6.8%), and Arg (6.2%). The length of the tRNA genes in *V. axillare* varied between 61 and 92 nucleotides, with the GC content ranging from 36.07% to 64.86%. The comparative analysis revealed four mutational hotspots with Pi values higher than 0.08 among the cp genomes of *V. axillare* and other Veroniceae species. They were composed of one protein-coding gene and three gene spacers with the highest Pi value region of *ndhF-rpl23*. Accordingly, the variation of the non-coding region was significantly higher than the conservative protein coding region, while the variation of both LSC and SSC regions was greater than that of the IR region. Therefore, our studies have shown that the Veroniceae plants have high mutation hotspots among different species, thus providing potential markers for plant identification and taxa investigation (Figure S1). Moreover, we identified 56 small single repeats in the cp genome of *V. axillare*, ranging from 10 to 18 bp.

The tribe Veroniceae contains 10 genera, of which *Veronica*, *Veronicastrum,* and *Lagotis* represent the largest genera containing over 300 plants, which were traditionally restricted to Scrophulariaceae. To further evaluate the phylogenetic relationships within the Veroniceae species, we performed the ML analysis based on the complete genome sequences of *V. axillare*, other representative Veroniceae species, Plantaginaceae, and Scrophulariaceae plants. The phylogenetic tree showed that *V. axillare* clustered together with *V. sibiricum* with high support value, thus suggesting a close genetic relationship between the two species (Figure 1). Additionally, the two Veroniceae species exhibited a sister relationship with the group of *Lagotis* plants, thereby providing evidence for further revising the classification within the tribe Veroniceae (Figure 1). The monophyletic group of *Veronica* species formed a clade with *Neopicrorhiza scrophulariiflora* with strong statistical support, thus indicating a close relationship between the two genera. Furthermore, since the combined group of Veroniceae plants was clustered with Scrophulariaceae rather than Plantaginaceae, further investigation on the taxonomy of tribe Veroniceae is required. Therefore, our results provide the basic genomic data for not only promoting the development of molecular markers, but also furthering the investigation on the conservation and breeding programs of the Veroniceae species.

**Figure 1.**
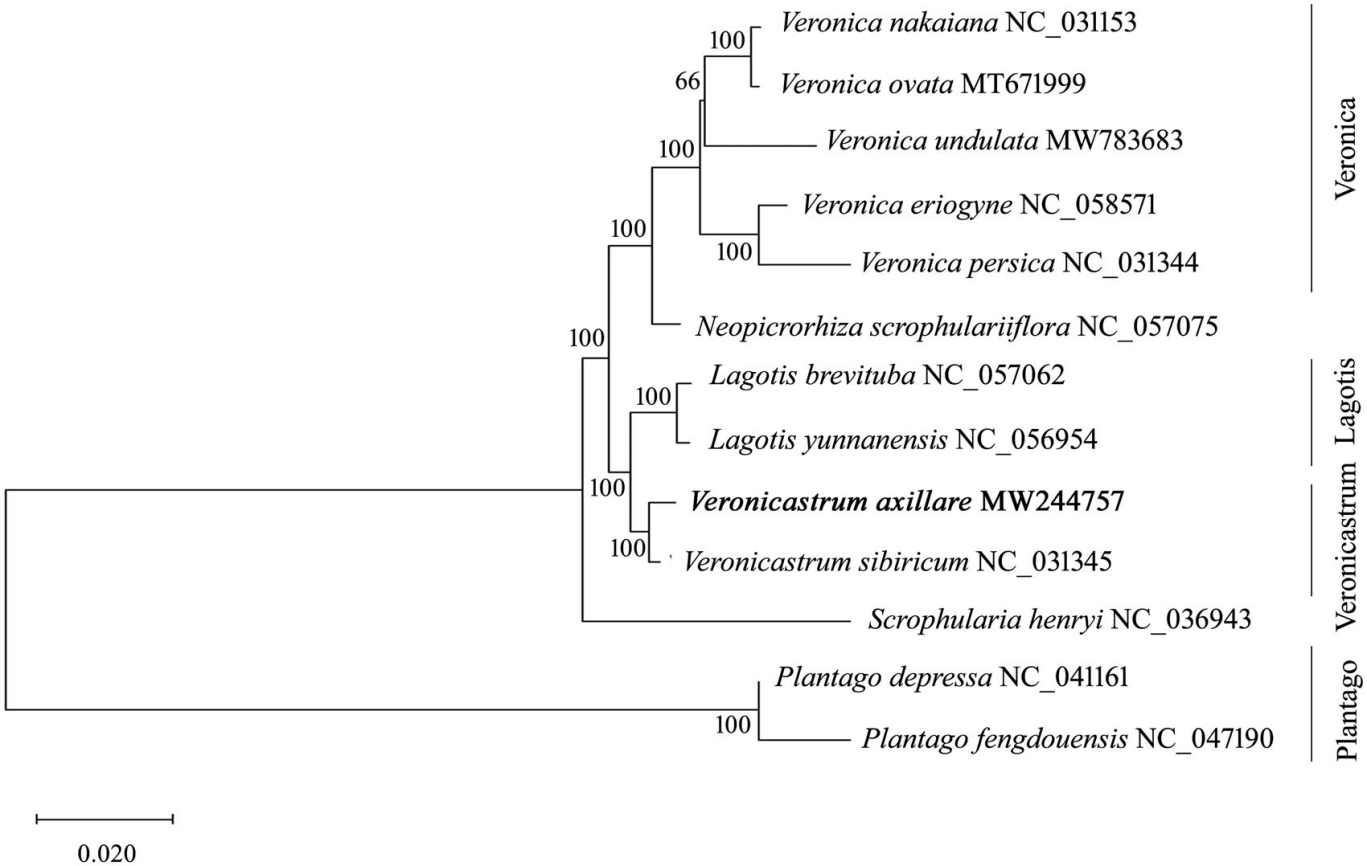
Phylogenetic tree reconstruction of the newly sequenced *Veronicastrum axillare* and other representative plants from the Veroniceae tribe, Plantaginaceae and Scrophulariaceae family. The tree was constructed using a maximum likelihood analysis by MAGE 7.0 based on the complete chloroplast genomes. The models of K2P and G + I were selected for maximum-likelihood analyses with 500 bootstrap replicates to calculate the bootstrap values. The newly determined chloroplast genome was marked with bold. The GenBank accession numbers were listed following the species name.

## Authors’ contributions

Jiawen Luo, Rubin Cheng and Yuqing Ge conceived and designed the experiments. Qiuyi Gong and Qiang Liu contributed to the resource sampling and species identification. Jiawen Luo and Qiuyi Gong performed the experiments. Jiawen Luo and Manjia Zhou analyzed the data and wrote the paper. Rubin Cheng and Yuqing Ge revised and approved the final version of the paper. All authors have reviewed and approved the manuscript.

## Ethics approval

Since the Project of Quality Guarantee System of Chinese Herbal Medicines, Dr. Rubin Cheng obtained the permission by Zhejiang Chinese Medical University to collect plant species from the mountain of Zhejiang Province. Because of the important medical value of *V. axillare*, Dr. Cheng collected the specimen of V. axillare for further molecular study. The plant material collection and experimental research were conducted according to the Plant Protection and Regulation of Zhejiang Chinese Medical University.

## Data Availability

The genome sequence data that support the findings of this study are openly available in GenBank of NCBI at (https://www.ncbi.nlm.nih.gov/) under the accession no. MW244757. The associated BioProject, SRA, and BioSample numbers of *Veronicastrum axillare* are PRJNA698722, SRR13607868 and SAMN17734771, respectively.
